# Impairments of intestinal arginine and NO metabolisms trigger aging-associated intestinal barrier dysfunction and ‘inflammaging'

**DOI:** 10.1016/j.redox.2022.102528

**Published:** 2022-10-31

**Authors:** Annette Brandt, Anja Baumann, Angélica Hernández-Arriaga, Finn Jung, Anika Nier, Raphaela Staltner, Dragana Rajcic, Christian Schmeer, Otto W. Witte, Barbara Wessner, Bernhard Franzke, Karl-Heinz Wagner, Amélia Camarinha-Silva, Ina Bergheim

**Affiliations:** a Department of Nutritional Sciences, R.F. Molecular Nutritional Science, University of Vienna, Vienna, Austria; b Institute of Animal Science, University of Hohenheim, Stuttgart, Germany; c Hans-Berger Department of Neurology, University Hospital Jena, Jena, Germany; d Centre for Sport Science and University Sports, University of Vienna, Vienna, Austria; e Department of Nutritional Sciences, University of Vienna, Vienna, Austria

**Keywords:** Aging, Endotoxin, Nitric oxide, Intestinal permeability, Microbiota

## Abstract

Aging is considered a state of low grade inflammation, occurring in the absence of any overt infection often referred to as ‘inflammaging'. Maintaining intestinal homeostasis may be a target to extend a healthier status in older adults. Here, we report that even in healthy older men low grade bacterial endotoxemia is prevalent. In addition, employing multiple mouse models, we also show that while intestinal microbiota composition changes significantly during aging, fecal microbiota transplantation to old mice does not protect against aging-associated intestinal barrier dysfunction in small intestine. Rather, intestinal NO homeostasis and arginine metabolism mediated through arginase and NO synthesis is altered in small intestine of aging mice. Treatment with the arginase inhibitor norNOHA prevented aging-associated intestinal barrier dysfunction, low grade endotoxemia and delayed the onset of senescence in peripheral tissue e.g., liver. Intestinal arginine and NO metabolisms could be a target in the prevention of aging-associated intestinal barrier dysfunction and subsequently decline and ‘inflammaging'.

## Highlights

• In humans ‘healthy' aging is associated with increased levels of TLR2 and TLR4 ligands in blood.
• ‘Healthy' aging is associated with an impaired intestinal barrier function in mice.
• NO metabolism rather than the intestinal microbiota seems to affect aging associated intestinal barrier dysfunction.

## Graphical abstract

**Figure:**
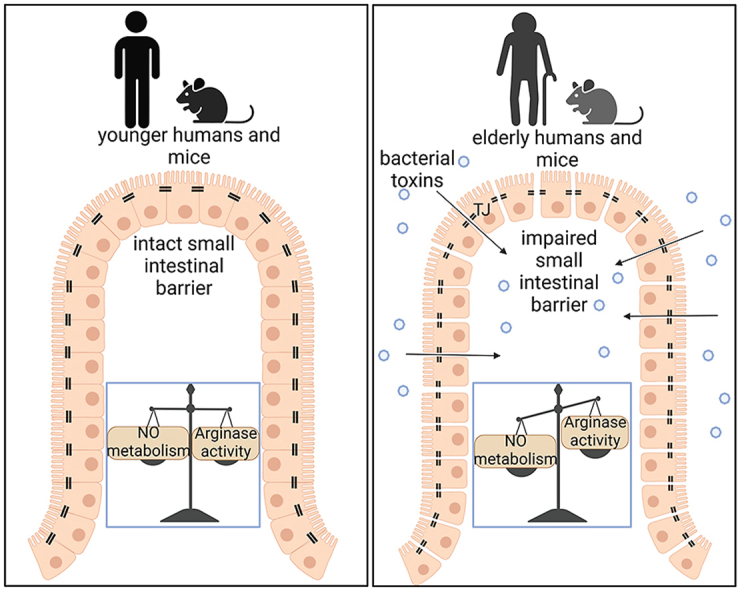

## Abbreviations:

3-NT: 3-nitrotyrosine
AB: antibiotics
Arg: arginase
Bmi1: B lymphoma Mo-MLV insertion region 1 homolog
CD14: cluster of differentiation 14
DFMO: difluoromethylornithine
EDTA: ethylenediaminetetraacetic acid
FMT: fecal microbiome transfer
iNOS: inducible nitric oxide synthase
KRH: Krebs-Henseleit-bicarbonate
LBP: lipopolysaccharide binding protein
Lrig1: leucine-rich repeats and immunoglobulin-like domains protein 1
mo: months
MOPS: 3-(*N*-morpholino) propanesulfonic acid
Muc2: mucin 2
MyD88: myeloid differentiation primary response 88
NO: nitric oxide
Lgr5: leucine-rich repeat-containing G-protein coupled receptor 5
norNOHA: N(ω)-hydroxy-nor-l-arginine
OTU: operative taxonomic unit
PAI-1: plasminogen activator inhibitor-1
PERMANOVA: Permutational analysis of variance
SDS: sodium dodecyl sulfate
SIMPER: Similarity percentages analysis
Tert: telomerase reverse transcriptase
TLR: toll-like receptor
TNFα: tumor necrosis factor alpha
ZO-1: zonula occludens-1

## 1 Introduction

Results of the Global Burden of Disease Study suggest that world-wide life expectancy at birth rose by 6.3 years from 67.2 years in 2000 to 73.5 years in 2019 and that this trend will sustain [1]. Studies also suggest that the expected healthy life span will not increase at the same rate but rather that time spend unhealthy will be expanded [1]. Despite intense research efforts throughout the last decades, mechanisms underlying aging-associated decline and also their diversity are not yet fully understood. In recent years, evidences from animal and human studies accumulated suggesting that intestinal homeostasis, and especially, mucosal integrity may be major factors for health and well-being (for overview see Refs. [2,3]). Indeed, together with a stable microbiota the mucus layer acts as first line of defense against external injuries (for overview see Refs. [3,4]), regulates entry and digestion of food-derived nutrients and forms and shapes the development of the immune system (for overview see Refs. [5,6]). While it has been suggested that the gross architecture of the intestinal epithelium in the small and large intestine is not markedly affected by aging [7,8], studies in rodents and non-human primates suggest that the number of goblet cells increases while expression of α-defensines, lysozyme and F4/80 mRNA expression, as well as NO_x_ levels and protein concentration of tight junction protein decreased with increasing age [[9], [10], [11], [12]]. These alterations were found to go along with an increase in intestinal permeability [10,13,14] and bacterial endotoxin levels [12,14,15]. Results of several studies further suggest that these aging-associated alterations are linked to changes of intestinal microbiota in older animals and humans [9,11,12,[16], [17], [18]]. It has been suggested that aging-associated intestinal microbiota dysbiosis and the increase of systemic tumor necrosis factor alpha (TNFα) levels found in old mice may be critical in aging-related intestinal barrier dysfunction [19]. However, whether and how intestinal microbiota or other alterations at the level of the gut affect intestinal barrier function in aging is not yet fully understood.

Starting from this background, we aimed to determine (1) whether changes in markers of intestinal integrity are also found in healthy older individuals and (2) molecular mechanisms possibly involved in the aging-associated changes in intestinal integrity.

## 2 Material and methods

### 2.1 Human subjects

Fasting plasma samples from 16 young, healthy, male adults (ages 23–33 years) and 16 healthy male older subjects (≥75 years of age) collected in the due course of screening visits for nutritional intervention studies in our department were analyzed. All studies were approved by the respective local ethics committees of the University of Vienna, Austria and were performed in accordance with the ethical standards laid down in the Helsinki Declaration of 1975 as revised in 1983. Studies are registered at http://www.clinicaltrials.gov (NCT01775111, NCT03482284, NCT04847193 and NCT04341818). Written informed consent was obtained from all subjects before the study. None of the subjects included in this analysis suffered from any of the following diseases: metabolic diseases e.g., cardiovascular diseases, type 2 diabetes, and non-alcoholic fatty liver disease, chronic inflammatory diseases, malignant diseases or took medication to control any of these diseases or were considered obese (BMI >20.0 to <30.0 kg/m^2^).

### 2.2 Mouse models

All procedures in mice were approved by local authorities and animals were handled in accordance with the European Convention for the Protection of Vertebrate Animals used for Experimental and Other Scientific Purposes (Thüringer Landesamt, Bad Langensalza, Germany and BMBWF-66.006/0014-V/3b/2019, BMBWF-66.006/0013-V/3b/2018, Vienna, Austria). Trial 1: To determine markers of intestinal permeability and to analyze intestinal microbiota, tissue and blood from portal vein was obtained from 3 and 24 months old C57BL/6J male mice anesthetized with nitrous oxide housed in a pathogen-free animal facility (Jena University Hospital) under standardized conditions. Tissues were either snap-frozen immediately or were fixed in neutral-buffered formalin for the histological staining. Blood was spun and plasma was obtained and frozen until further use.

Trial 2: To determine the effects of fecal microbiome transfer (FMT) on aging-associated intestinal barrier decline, feces were collected from 3 months old male C57BL/6J mice and 17 months old male C57BL/6J mice housed in groups in a specific pathogen-free animal facility at the University of Vienna under standardized conditions. The latter mice showed signs of intestinal barrier dysfunction e.g., significantly higher bacterial endotoxin levels in peripheral blood obtained from the *vena fascialis* and increased markers of senescence e.g., higher p16 protein levels in blood (see Fig. 3). Prior to the FMT treatment, naïve 17 months old mice were treated with an antibiotic mixture (polymyxin B (92 mg/kg BW) and neomycin (216 mg/kg BW)) in drinking water for three days. Mice were then randomly assigned to two groups treated with fecal microbiota from 1) young (o + yFMT) or 2) from old mice (o + oFMT), by oral gavage three times weekly for the following six weeks. Feces used for FMT were collected and stored as previously described [20] and microbiota composition was analyzed as detailed below.

Trial 3: To determine the role of NO synthesis and arginase activity in aging-associated decline of intestinal barrier function, old male C57BL/6J mice (age 17 months) showing signs of intestinal barrier dysfunction and old age e.g., elevated bacterial endotoxin levels and markers of senescence in blood were either treated with the arginase inhibitor N(ω)-hydroxy-nor-l-arginine (norNOHA) (Bachem, Switzerland, 10 mg/kg BW, i.p.) or vehicle (NaCl, i.p.) three times weekly for six weeks. Mice were kept in groups in a specific pathogen-free animal facility at the University of Vienna under standardized conditions. At the end of trials 2 and 3 animals were anesthetized with ketamine (100 mg/kg BW) and xylazine (16 mg/kg BW), and after blood was drawn from the portal vein, mice were sacrificed, and tissue was collected.

### 2.3 Everted gut sac and xylose permeation measurement

Everted gut sacs were built of rinsed small intestinal tissue as previously described in detail [21]. In brief, after everting and filling the tissue sacs with 1x Krebs-Henseleit-bicarbonate buffer with 0.2% BSA (KRH), tissue sacs were incubated in gassed KRH buffer (95% O_2_/5% CO_2_) at 37 °C supplemented with xylose (0.1% (w/v) for either 5 min to determine intestinal permeability or supplemented for 55 min with 1 μM of arginase inhibitor norNOHA or 20 μM ROCK-inhibitor Y-27632 or 1 mM difluoromethylornithine (DFMO). The latter experiments were followed by a 5 min incubation with additional xylose (0.1% (w/v)) to measure xylose permeation. Xylose concentration was measured using a modified protocol for measuring xylose in urine and serum samples based on phloroglucinol as previously published [21,22], and expressed as μmol/cm. Concentrations of Y-27632, norNOHA and DFMO were determined in pilot studies. The remaining tissue was snap-frozen.

### 2.4 Assessment of toll-like receptor 2 (TLR2) and TLR4 ligands

Limulus amebocyte lysate assay (Charles River, France) was used for detecting endotoxin concentration in portal and peripheral plasma of mice and in plasma of patients, as described previously [23]. In addition, commercially available SEAP reporter HEK293 cells assays (Invivogen, USA) were used to determine total TLR2 and TLR4 ligands in human as well as mouse plasma as detailed previously [24].

### 2.5 ELISA and arginase activity

Lipopolysaccharide binding protein (LBP) and plasminogen activator inhibitor-1 (PAI-1) protein levels were measured in peripheral plasma of humans and plasma from portal vein of mice using commercially available ELISAs (Abnova, Taiwan and LOXO, Germany), respectively, as detailed by the manufacturer. Arginase activity in proximal small intestine was measured, as previously described [21].

### 2.6 Evaluation of goblet cell number

Paraffin-embedded intestinal tissue sections (4 μm) from proximal and distal small intestine and colon were stained with Alcian Blue and periodic acid-Schiff staining, as previously described by others [25]. Number of cells/100 μm villus was assessed in proximal and distal small intestine as well as colon using a microscope with an integrated camera (DFC 450 C Leica, Germany).

### 2.7 Immunohistochemical staining of 3-nitrotyrosine (3-NT) and tight junction proteins occludin and zonula occludens-1 (ZO-1) as well as arginase-1 (Arg-1) and -2 (Arg-2) in intestinal tissue

Paraffin-embedded intestinal tissue sections (4 μm) were stained for 3-NT, occludin and ZO-1 as well as Arg-1 and -2 as previously described [21,26,27]. In brief, sections were incubated with specific primary antibodies (3-NT: Santa Cruz Biotechnology, USA; occludin and ZO-1: Invitrogen, USA; arginase-1 and arginase-2: Cell Signaling, USA) following peroxidase-linked secondary antibody and diamino-benzidine to determine specific binding. The extent of occludin and ZO-1 staining was defined as the percentage of microscopic field within the default color range capturing eight pictures per sample using a microscope with an integrated camera (Leica DM6B, Leica DMC4500, Leica, Germany). 3-NT-positive cells were counted per mm villus in eight randomly selected microscopic fields per sample.

### 2.8 RNA isolation and real-time RT-PCR

Total RNA was extracted from snap-frozen liver, proximal and distal small intestinal as well as colonic tissue using peqGOLD TriFast (Peqlab, Germany) and reverse transcribed as described previously [28]. mRNA expression of genes listed in Supplemental Table 1 were determined by real-time RT-PCR as described previously [29].

### 2.9 Western blot

To detect Arg-1, Arg-2, pROCK2, ROCK2, p16 (CDKN2A) and cluster of differentiation 14 (CD14) by Western blot, protein was either isolated from snap-frozen small intestine using RIPA buffer (20 mM 3-(*N*-morpholino) propanesulfonic acid (MOPS), 150 mM NaCl, 1 mM ethylenediaminetetraacetic acid (EDTA), 1% Nonidet P-40 and 0.1% sodium dodecyl sulfate (SDS)) containing protease and phosphatase inhibitor cocktails (Sigma-Aldrich, Germany) or plasma samples were used which were diluted with loading buffer (0.3 M Tris, 10% SDS, 50% glycerol, 0.05% bromphenol blue, 20% β-mercaptoethanol). Samples (10–30 μg protein/lane) were separated in a SDS-polyacrylamide gel and transferred to an Immun-Blot®-polyvinylidene difluoride membrane (Bio-Rad, USA). Resulting membranes were incubated with specific primary antibodies (Arg-1, Arg-2: Cell Signaling, USA; ROCK2, pROCK2: GeneTex, USA; CD14: Santa Cruz, USA; p16 (CDKN2A): biorbyt, UK) and corresponding secondary antibodies. Protein bands were detected using a luminol-based enhanced chemiluminiscence HRP substrate (Super SignalWest Dura kit, Thermo Fisher Scientific, USA) and analyzed densitometrically (ChemiDoc MP System, Bio-Rad, USA). Band intensities of proteins determined in tissue were normalized to β-actin protein bands while those determined in plasma samples were normalized to ponceau staining as detailed before [26].

### 2.10 Detection of nitrite

Nitrite levels in scraped mucosa and small intestinal tissue were determined using a commercially available Griess reagent kit according to the instructions of the manufacturer (Promega, USA) and as detailed before [21]. In brief, tissue samples were homogenized in PBS, centrifuged and Griess reagent was added to the resulting supernatant.

### 2.11 Illumina amplicon sequencing of microbiota from small intestinal content

Total DNA was extracted from small intestinal content of mice using the FastDNA™ SPIN Kit for soil from MP Biomedicals (Solon, OH, USA), following the manufacturer's instructions. Illumina library preparation was performed according to Hernandez-Arriaga et al. [30] targeting the V1-2 region of the 16S rRNA gene. Samples were sequenced using 250 bp paired-end sequencing chemistry on an Illumina MiSeq platform. Raw reads were quality filtered, assembled and aligned using Mothur pipeline [31]. A total of 24,890 ± 13,365 raw reads were obtained per sample. UCHIME was used to find possible chimeras and reads were clustered at 97% identity. Only operative taxonomic units (OTUs) present on average abundance higher than 0.0001% and with a sequence length higher than 250 bp were considered for further analysis. The closest representative was manually identified with seqmatch from RDP as described previously [32]. Sequences were submitted to European Nucleotide Archive under the accession number PRJEB52291.

### 2.12 Statistical analysis

All values are shown as means ± sem. Outliers were identified using Grubb's test. Differences between two groups were determined using an unpaired two-tailed students t-test, after testing for normal distribution, while for three groups a one-way ANOVA was performed (Graph Pad Prism Version 7.0, USA). Differences were considered statistically significant when *p* < 0.05.

Regarding Illumina amplicon sequencing data, a sample similarity matrix was created using Bray-Curtis coefficient [33] and further explored with Principal Coordinates Analysis (PCoA) [34]. Permutational analysis of variance (PERMANOVA) was used to evaluate statistical differences between different groups. Similarity percentages analysis (SIMPER) was used to identify the OTUs that contribute to the differences between groups (PRIMER-E, version 7.0.9, Plymouth Marine Laboratory, Plymouth, UK) [35]. Differences in the abundance of OTUs of interest between age groups were evaluated using the non-parametric Wilcoxon test [36]. Visualizations were created using R version 4.1.0 with R Studio and Calypso [37,38].

## 3 Results

### 3.1 Markers of intestinal permeability in young and old human male subjects

**Fig. 1:**
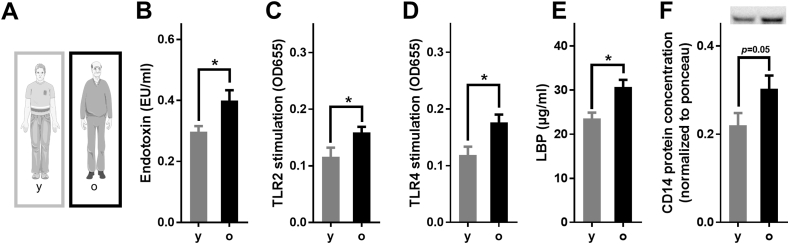
**Effect of aging on markers of intestinal permeability in young and old male healthy humans.** (A) Study design, (B) endotoxin concentration and (C) levels of toll-like receptor 2 (TLR2) and (D) TLR4 ligands, (E) lipopolysaccharide binding protein (LBP) concentration and (F) cluster of differentiation 14 (CD14) concentration in plasma of young and old humans (n = 15–16). Values are means ± sem. *p < 0.05 due to unpaired two-tailed students t-test. mo: months.

While not showing any signs of frailty or suffering from any overt diseases and only being overweight but not obese (Supplemental Table 2), bacterial endotoxin and TLR2 ligand levels in the peripheral blood of male older adults were all significantly higher than in young male adults (Fig. 1). LBP and soluble CD14 protein levels in plasma of older men were also higher than in young men.

### 3.2 Markers of intestinal permeability and intestinal morphology in healthy young and old male mice

**Fig. 2:**
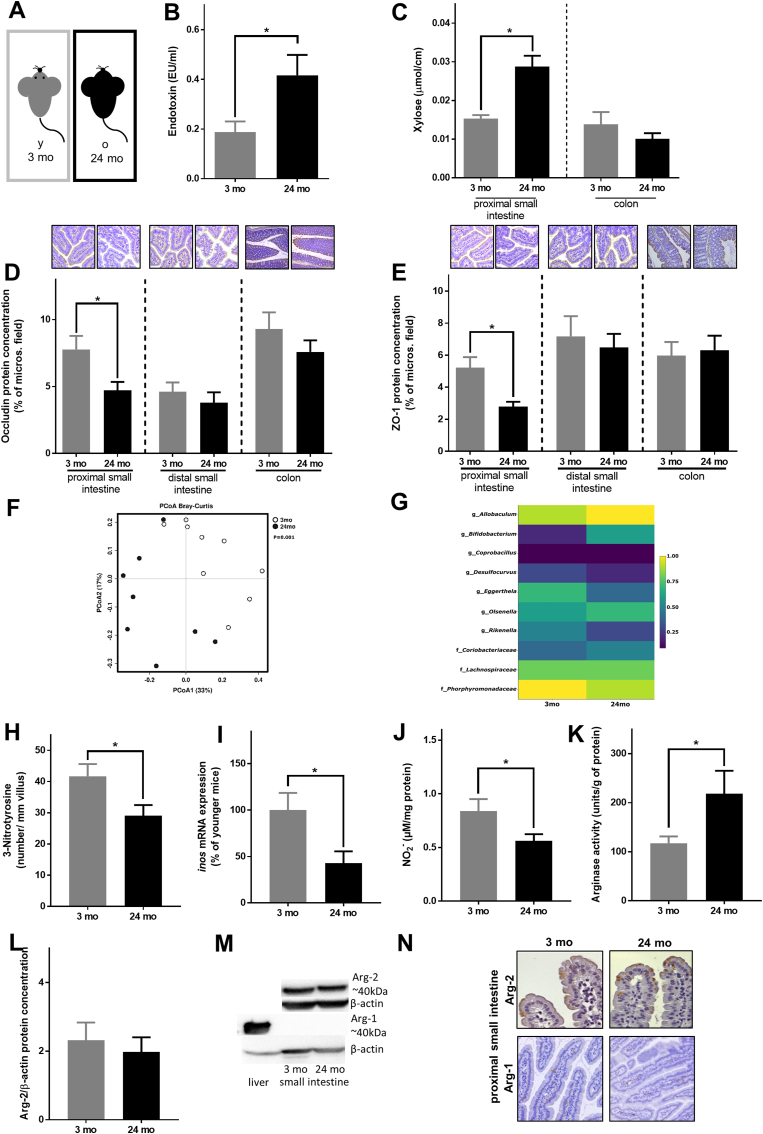
**Effect of aging on markers of intestinal permeability, NO metabolism and arginase activity in intestine of young and old male healthy mice.** (A) Study design, (B) endotoxin concentration in plasma of portal vein and (C) xylose permeation of everted sacs of 3 months and 24 months old mice. Densitometric analysis and representative pictures (small intestine: 400x, colon: 100x) of (D) occludin as well as (E) zonula occludens-1 (ZO-1) in intestine of young and old mice. (F) PCoA plot showing the microbial communities of each sample and (G) significant microbial communities at genus and family level. (H) Evaluation of 3-nitrotyrosine (3-NT) positive cells; (I) *inducible nitric oxide synthase* (*inos*) mRNA expression and (J) NO_2_^−^ concentration in proximal small intestine. (K) Arginase activity and (L) densitometric analysis of arginase-2 Western blot as well as (M) representative Western blots and (N) representative pictures of arginase-1 (200x) and arginase-2 (630x) protein staining of proximal small intestine of 3 months and 24 months old mice (n = 5–9). Values are means ± sem. *p < 0.05 due to unpaired two-tailed students t-test. mo: months.

In line with the findings in humans and despite being fed the same diet, male old mice were significantly heavier and markers of senescence [[39], [40], [41]], e.g. PAI-1 protein levels in plasma and *p1*6 mRNA expression of hepatic tissue (Table 1) were significantly higher than in young animals. Portal bacterial endotoxin concentration was significantly higher in 24 months old mice than in young animals (+2.2-fold, Fig. 2) and was associated with an increased xylose permeation in the proximal small intestine but not in colon. While morphology and number of goblet cells were similar in proximal and distal small intestine as well as in colon in old and young mice (Supplemental Fig. 1, Supplemental Table 3), protein and mRNA expression levels of the tight junction proteins occludin and ZO-1 were significantly lower in proximal small intestine of old mice than in young animals (Fig. 2, Supplemental Fig. 1). Similar differences were not found in distal small intestine and colon (Fig. 2, Supplemental Fig. 1). Since the most prominent changes in tight junction protein levels were found in the proximal small intestine, all further studies were focused on this part of the small intestine.

**Table 1:** Effect of aging on markers of senescence, antimicrobial peptides and intestinal stem cells in mice.

	3 months	24 months
**Body weight (g)**	30.2 ± 0.4	34.3 ± 1.1*
**PAI-1 (ng/ml)**^**ß**^	1.86 ± 0.22	3.31 ± 0.39*
***p1*6 mRNA**^**+**^	100 ± 9.6	2,571 ± 878*
***Lysozyme* mRNA**^**#**^	100 ± 36	13.2 ± 3.7*
***Cramp* mRNA**^**#**^	100 ± 9.8	68.3 ± 8.9*
***Bmi1* mRNA**^**#**^	100 ± 23	96.9 ± 16
***Lgr5* mRNA**^**#**^	100 ± 19	43.2 ± 6.4*
***Tert* mRNA**^**#**^	100 ± 9.2	71.7 ± 4.5*
***Lrig1* mRNA**^**#**^	100 ± 20	54.3 ± 7.5

Values are means ± sem. mRNA results are shown as % of younger mice. *p < 0.05 due to unpaired two-tailed students t-test, ^**ß**^assessed in plasma, ^+^assessed in hepatic tissue, ^#^assessed in proximal small intestine (n = 6–9). PAI-1: plasminogen activator inhibitor 1; Cramp: Cathelicidin-related antimicrobial peptide; Bmi1: B lymphoma Mo-MLV insertion region 1 homolog; Lgr5: Leucine-rich repeat-containing G-protein coupled receptor 5; Tert: telomerase reverse transcriptase; Lrig1: Leucine-rich repeats and immunoglobulin-like domains protein 1.

**Fig. 3:**
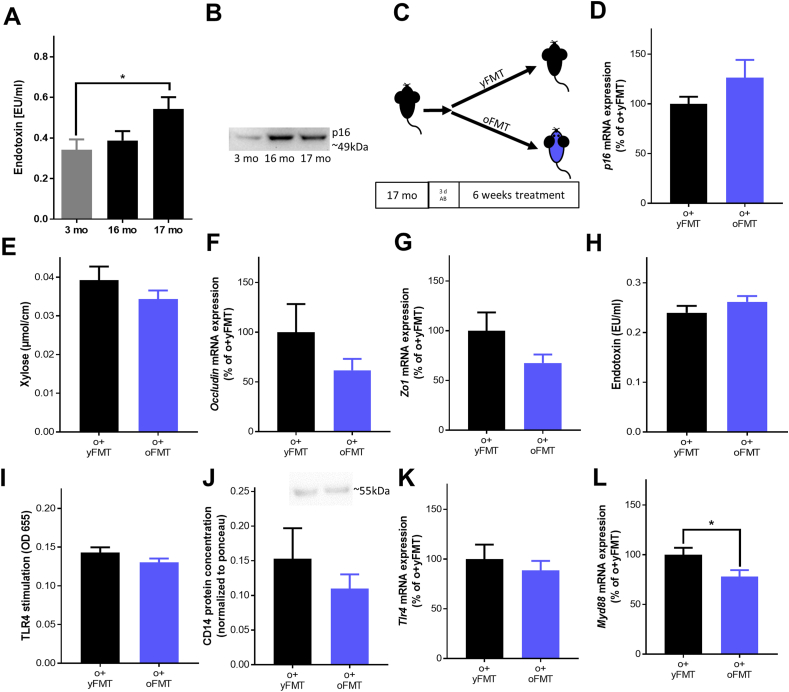
**Effect of FMT on markers of intestinal barrier function in mice.** (A) Endotoxin concentration in *vena fascialis* of mice from SPF facility at different age (n = 5–9; *p < 0.05 due to one-way ANOVA) as well as (B) representative blot of p16 protein in plasma. (C) Study design, (D) hepatic *p1*6 mRNA expression, (E) xylose permeation of everted gut sac, (F) *occludin* and (G) *zonula occludens-1* (*Zo1*) mRNA expression of proximal small intestine, (H) endotoxin concentration, (I) levels of toll-like receptor 4 (TLR4) ligands (J) and densitometric analysis of cluster of differentiation 14 (CD14) Western blot in plasma from portal vein, (K) *Tlr4* and (L) *Myeloid differentiation primary response 88* (*Myd88*) mRNA expression of hepatic tissue of old (o) mice receiving young and old FMT (+yFMT, +oFMT), (n = 7–10). Values are means ± sem. *p < 0.05 due to unpaired two-tailed students t-test. AB: antibiotics, mo: months, FMT: fecal microbiota transfer.

### 3.3 Antimicrobial peptides, intestinal stem cell markers and intestinal microbiota composition in the proximal small intestine of young and old mice

The loss of tight junction proteins in the proximal small intestine was associated with significantly lower mRNA expression of the antimicrobial peptides *Lysozyme* and *Cramp* in old mice when compared to young animals. (*Lysozyme* and *Cramp*: *p* < 0.05; Table 1). Expressions of stem cell markers *Leucine-rich repeat-containing G-protein coupled receptor 5* (*Lgr5)* and *telomerase reverse transcriptase* (*Tert)* were significantly lower in proximal small intestine of old mice compared to younger animals, while *Leucine-rich repeats and immunoglobulin-like domains protein 1* (*Lrig1)* and *B lymphoma Mo-MLV insertion region 1 homolog* (*Bmi1)* mRNA expressions were not different between groups (Table 1).

The total microbial community of the small intestine was statistically different between young and old mice (*p* = 0.001, Fig. 2F), where 28.6% of the OTU were unique for young mice and 9% for old mice. The average dissimilarity between groups was 60%. Analysis of the community structure showed that Firmicutes were more abundant in old mice than in young mice (*p* = 0.07). The abundances of the genera *Allobaculum* and *Bifidobacteria* were higher in old mice when compared to young mice (*p* < 0.05 for *Allobaculum* and *Bifidobacteria* abundance). At genus level, unclassified members of *Porphyromonadaceae* and *Lachnospiraceae* were significantly more abundant in small intestine of young than in old animals (*p* < 0.05) (Fig. 2, Supplemental Table 4).

### 3.4 Markers of NO homeostasis in small intestine of old mice

Alterations of NO-homeostasis have been discussed to be critical in the development of intestinal barrier dysfunction (for overview see Ref. [42]). Expression of *inducible nitric oxide synthase* (*inos)* mRNA, concentration of 3-NT protein adducts and NO_2_^−^ in small intestinal tissue were significantly lower in 24 months old mice than in 3 months old animals (Fig. 2, Supplemental Fig. 1). While protein levels of arginase-2, the isoform of arginase predominately found in the tip of the villi in small intestine, were similar between young and old mice, the activity of total arginase was significantly higher in small intestinal tissue of old mice when compared to young animals (Fig. 2).

### 3.5 Intestinal barrier function and markers of senescence in FMT-treated old male mice

Microbiota composition in fecal samples used for the FMT differed markedly between young and old donors (Supplemental Fig. 2); however, the six week long FMT had no effects on gross signs of aging e.g., body weight, activity or general behavior of 17 months old mice showing signs of beginning senescence and impaired barrier function before the treatment. Expression of *p1*6 mRNA in liver tissue and PAI-1 concentration in plasma were similar between groups after the FMT. Also, neither intestinal permeability in small intestine nor expression of tight junction proteins or bacterial endotoxin and sCD14 protein levels in plasma differed between treatment groups (Fig. 3, Supplemental Table 5). Furthermore, *Tlr4* mRNA expression in liver tissue was similar between groups, while in o + oFMT-treated mice, *Myeloid differentiation primary response 88 (Myd88)* mRNA expression was significantly lower than in o + yFMT-treated animals (Fig. 3). Expression of *Mucin 2* (*Muc2)*, *Lysozyme* and stem cell factors *Lgr5* and *Tert* mRNA in small intestinal tissue was also similar between groups regardless of microbiota transplanted (Supplemental Table 5). However, the microbiota composition of o + oFMT- and o + yFMT-treated mice was significantly different (Supplemental Fig. 2 and Supplemental Table 6).

### 3.6 Intestinal barrier function and markers of senescence in male old mice treated with an arginase inhibitor

**Fig. 4:**
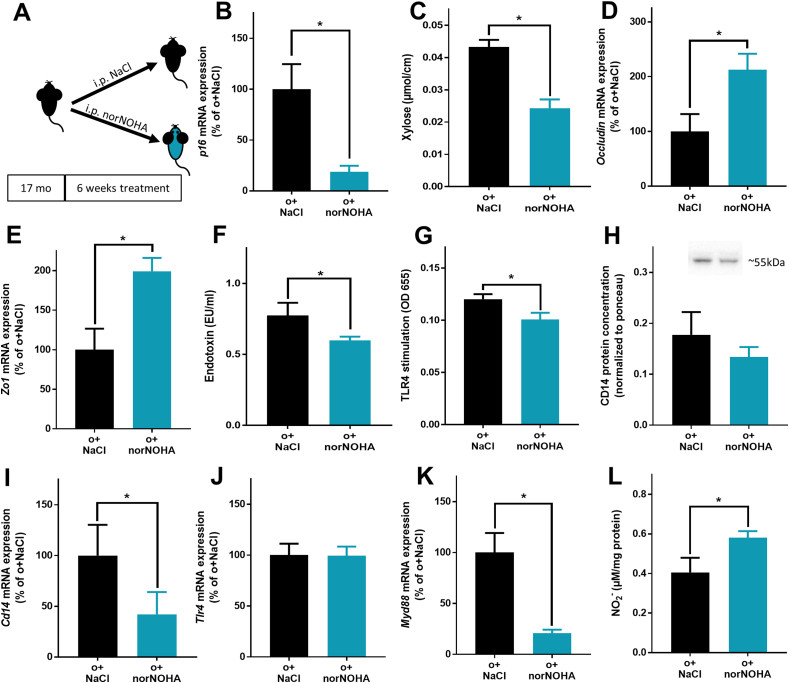
**Effect of arginase inhibition on markers of intestinal barrier in proximal small intestine of mice.** (A) Study design, (B) hepatic *p1*6 mRNA expression, (C) xylose permeation in everted gut sacs and (D) *occludin* and (E*) zonula occludens-1* (*Zo1*) mRNA expression in proximal small intestine, (F) endotoxin concentration, (G) levels of toll-like receptor 4 (TLR4) ligands in plasma of portal vein, (H) cluster of differentiation 14 (CD14) concentration in plasma determined by Western blot, (I) *Cd14*, (J) *Tlr4* and (K) *Myeloid differentiation primary response 88* (*Myd88*) hepatic mRNA expression and (L) NO_2_^−^ concentration of scraped mucosa of intestinal tissue in old (o) mice treated with NaCl or N(ω)-hydroxy-nor-l-arginine (norNOHA) (n = 5–7). Values are means ± sem. *p < 0.05 due to unpaired two-tailed students t-test. mo: months.

In 17 months old mice, the treatment with the arginase inhibitor norNOHA for 6 weeks resulted in significantly lower p16 mRNA expression in hepatic tissue, lower intestinal permeability, higher mRNA expression of tight junction proteins *occludin* and *Zo1* in proximal small intestine, lower bacterial endotoxin in plasma and lower *Cd14* as well as *Myd8*8 mRNA expression in liver tissue when compared to age matched vehicle treated mice (Fig. 4). *Tlr4* mRNA expression in liver tissue and CD14 and PAI-1 concentration in plasma were similar between groups. Also, levels of NO_2_^−^ were higher in small intestinal tissue of norNOHA-treated mice compared to vehicle controls (Fig. 4). Furthermore, mRNA expression of stem cell markers *Lgr5* and *Tert* were significantly higher in small intestinal tissue of norNOHA-treated mice compared to vehicle-treated mice (Table 2). No differences were found between groups when assessing ZO-1 protein staining and mRNA expression of *Lysozyme*, *Muc2* as well as the total bacterial microbiota composition in small intestine (Table 2, Supplemental Fig. 3 and Supplemental Table 7).

**Table 2:** Effect of 6 weeks treatment with norNOHA on body weight, markers of senescence and intestinal permeability, antimicrobial peptides and stem cell markers in old mice.

	o+ NaCl	o+ norNOHA
**Body weight (g)**	33.8 ± 0.7	31.5 ± 1.1
**PAI-1 (ng/ml)**	1.4 ± 0.3	1.9 ± 0.1
**ZO-1 protein**^***#***^	3.2 ± 0.3	3.7 ± 0.5
***Muc2* mRNA**	100 ± 35	56.3 ± 13
***Lysozyme* mRNA**	100 ± 33	139 ± 23
***Lgr5* mRNA**	100 ± 22	173 ± 15*
***Tert* mRNA**	100 ± 20	178 ± 15*

Values are means ± sem. mRNA results are shown as % of o + NaCl and detected in small intestinal tissue, while PAI-1 levels were detected in plasma. *p < 0.05 due to unpaired two-tailed students t-test (n = 6–7), ^#^densitometric analysis of protein staining. PAI-1: plasminogen activator inhibitor 1; ZO-1: zonula occludens-1; Muc2: mucin 2; Lgr5: Leucine-rich repeat-containing G-protein coupled receptor 5; Tert: telomerase reverse transcriptase. norNOHA: N(ω)-hydroxy-nor-l-arginine; o: old.

### 3.7 Effect of inhibition of arginase/ROCK signaling cascade in everted gut sac

**Fig. 5:**
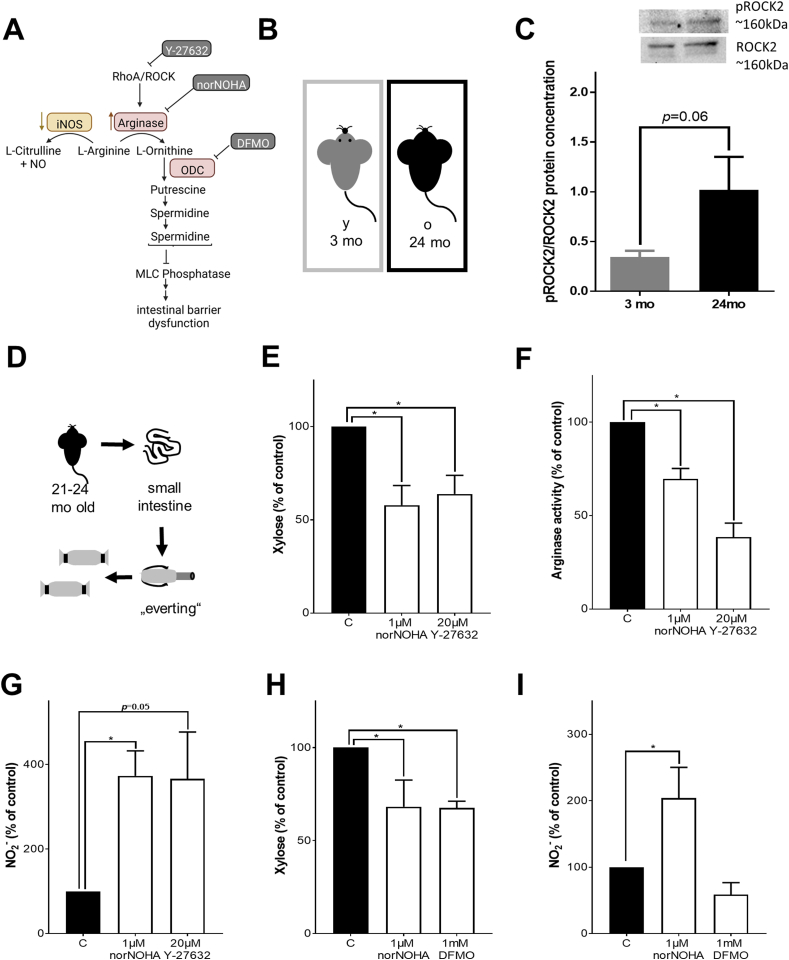
**Effect of inhibition of arginase and ROCK signaling cascade in old everted gut sacs.** (A) arginase signaling cascade, (B) study design, (C) pROCK2/ROCK2 Western blot in proximal small intestine of 3 months and 24 months old mice (n = 5–6, *p* = 0.06 due to unpaired two-tailed students t-test). (D) Schematic explanation of everted gut sacs, (E) Xylose permeation of everted gut sacs, (F) arginase activity and (G) NO_2_^−^ concentration in tissue of everted gut sacs from 21 to 24 months old mice incubated with KRH buffer (C), 1 μM N(ω)-hydroxy-nor-l-arginine (norNOHA) or 20 μM Y-27632 (n = 4, *p < 0.05 due to one-way ANOVA). (H) Xylose permeation of everted gut sacs, (I) NO_2_^−^ concentration in tissue of everted gut sacs from 21 to 24 months old mice incubated with KRH buffer (C), 1 μM N(ω)-hydroxy-nor-l-arginine (norNOHA) or 1 mM difluoromethylornithine (DFMO) (n = 3–5, *p < 0.05 due to one-way ANOVA). Values are means ± sem. mo: months.

To determine if an altered ROCK2 activity might be critical in the above described alterations, we determined phosphorylation of ROCK2 in small intestinal tissue of 3 and 24 months old mice. In small intestinal tissue of old mice, pROCK2 showed a trend towards higher levels than in young mice (*p* = 0.06, Fig. 5). Treatment of small intestinal everted sacs of old mice with the ROCK-inhibitor Y-27632 and norNOHA, respectively, decreased both permeability and arginase activity to a similar extend. These changes were associated with an increase in NO_2_^−^ levels in tissue (Fig. 5). Moreover, inhibiting ornithine decarboxylase with difluoromethylornithine (DFMO) resulted in a decreased permeability, too, while NO_2_^−^ levels were not affected.

## 4 Discussion

Results of recent studies suggest that aging is related to changes in intestinal microbiota composition and barrier function (for overview see Ref. [43]). It has also been discussed that changes in intestinal microbiota composition and/or the increased permeation of bacterial (endo)-toxin may enhance aging-associated decline. Here, employing studies in healthy older men and mice, we found that even in the absence of any overt health impairments, old age is associated with increased levels of bacterial (endo)-toxins and significant changes in intestinal microbiota composition, a loss of tight junction proteins and increased permeability in small intestine in old mice. Furthermore, expressions of antimicrobial peptides and several stem cell factors were also lower in small intestinal tissue while *Tlr4* mRNA expression was higher in the liver of old mice, further supporting the hypothesis that old age in mice is associated with impairments of intestinal barrier function in small intestine and an increased translocation of bacterial toxins. Interestingly, neither tight junction protein levels nor intestinal permeability were altered in large intestine of animals. Old age has been reported before to be associated with marked changes in diversity and the prevalence of specific bacterial strains in fecal and small intestinal microbiota, as well as elevated levels of bacterial endotoxin in a variety of species [9,12,[14], [15], [16], [17],^44^]. In baboons, these alterations have been linked to a loss of tight junction proteins in the large intestine [10], while in rodents, being in line with our finding, tight junctions’ protein levels were lower in small intestinal tissue [11]. However, data regarding changes of intestinal permeability in humans is still contradictory. Indeed, in studies employing sugar-based functional tests e.g., lactulose/mannitol or sucrose excretion, no differences or even lower intestinal permeability were reported in older adults compared to young healthy individuals [[45], [46], [47]]. It has been speculated that this apparent discrepancy between studies may be related to impairments of kidney function or a concomitant intake of drugs and prevalence of diseases in study subjects as well as their specific age (e.g. >75 year or 65–75 years of age) and their gender [45,46] and permeability tests used (e.g. aggregates of bacterial endotoxin with sizes up to 1,000 kDa [48,49] vs. markedly smaller molecular sizes of sugars employed in permeability tests (for overview see Ref. [50])). Taken together, further studies are needed to delineate changes in intestinal barrier function in aging humans. Still, results of the present study suggest that regardless of the species, old age is associated with elevated bacterial (endo)-toxin levels, and that in mice, these increases are related to changes of intestinal microbiota composition and a loss of tight junctions in the small intestinal tissue. These results by no means preclude that other aging-related alterations like an impaired clearance of bacterial endotoxin or elevated bioavailability in the gut might have been critical herein, too. Also, further studies are needed to assess if alterations alike are also found in women and female mice as in the present study only male subjects and mice were enrolled. Indeed, results of studies in *drosophila* suggest sex difference in the development of aging-associated intestinal degeneration [51].

### 4.1 Microbiota transfer is not associated with protection from changes in intestinal barrier function of mice

Previous studies by others (Thevaranjan et al., [19]) have suggested that the systemic increase in proinflammatory cytokines and aging-related impairments of intestinal barrier function are related to a diminished diversity of microbial community. In the same study it was also shown that co-housing old and young germ-free mice with old SPF-mice did result in similarly increased paracellular intestinal permeability. In contrast, young germ-free mice co-housed with old SPF-mice showed an increased intestinal permeability compared to young germ-free mice co-housed with young SPF-mice [19]. Furthermore, Thevaranjan et al. also reported, that germ-free mice showed lower intestinal permeability and mortality up to the age of 22 months. This was also associated with lower levels of plasma cytokines and subsequently with reduced ‘inflammaging' compared to age-matched old wild-type mice [19] further suggesting that intestinal microbiota and/or bacterial (endo-) toxins may be critical in the development of aging-associated decline. Fransen et al. showed that the transfer of bacteria from old mice to young germ-free mice promoted inflammation in the small intestine and increased intestinal permeability [52]. These data are partially in contrast with the findings of the present study where neither the transfer of fecal microbiota obtained from young to old mice nor form old to old mice affected intestinal permeability, markers of senescence, tight junction, or stem cell factor mRNA expression in the small intestine. Interestingly, microbiota composition in small intestine was significantly different between old mice receiving young FMT and old mice receiving old FMT. Differences between our studies and those of others might have resulted from marked differences in experimental design (germ-free mice vs. SPF animals, application of microbiota). Taken together, these data suggest that aging-associated intestinal barrier function may not only depend on changes of intestinal microbiota associated with aging but that other ‘independent' factors may be critical herein. However, these findings by no means preclude that intestinal microbiota may be critical in healthy aging but rather suggest that other cellular alterations - independent of intestinal microbiota - may impact aging-associated intestinal barrier dysfunction (see below).

### 4.2 Disturbances of intestinal NO homeostasis are critical in aging-associated intestinal barrier dysfunction

Intestinal barrier dysfunction has also been discussed to be related to an imbalance of iNOS and arginase activity [42,53,54]. Indeed, we have shown that the loss of intestinal barrier function in diet-induced non-alcoholic fatty liver disease is associated with an increased formation of NOx and lower activity of arginase [21,53]. In humans and rodents with inflammatory bowel disease, contrasting the findings in settings of metabolic disease, arginase-1 expression in intestinal tissue was reported to be induced and a genetic deletion of arginase-1 in immune cells was reported to arbitrate a significant protection against intestinal inflammation [55]. In the present study, old age in mice was associated with lower NOx levels in small intestinal tissue, while arginase activity was higher. Furthermore, the treatment with the arginase inhibitor norNOHA improved intestinal barrier function going along with lower bacterial endotoxin levels in portal plasma and a decreased expression of markers of senescence in liver while NOx levels in small intestinal tissue were higher. If treating mice with a NO donor to compensate the imbalance between NO synthesis and arginase activity during aging-associated impaired intestinal barrier exerts similar beneficial effects, remains to be determined. Also, if an oral supplementation of the amino acid arginine, shown to be a substrate for arginase but also allosteric regulator [53,56], affects aging-associated intestinal barrier dysfunction remains also to be determined in further experiments.

Somewhat in line with our findings, Xiong et al. suggest that a genetic deletion of arginase-2 attenuates the onset of senescence and extends life-span in mice [57]. In the present study, arginase-2 was the predominant isoform of arginase detected in the villi of the small intestine. Interestingly, while the expression of antimicrobial peptides and stem cell factors were higher in old mice treated with norNOHA, intestinal microbiota composition was similar between groups. These results suggest that while having been associated with the development of several aging-associated dysfunctions, maintaining intestinal barrier function in aging may not (solely) depend on intestinal microbiota. Rather alterations of cellular metabolism and herein especially of the NO homeostasis in small intestinal tissue might be critical in maintaining intestinal barrier function, too. Interestingly, results employing *ex vivo* models further suggest that the induction of arginase activity is related to changes in the phosphorylation of ROCK2 and the downstream signaling cascade of arginase. The RhoA/ROCK signaling cascade has been indicated to regulate both, arginase-2 and myosin light chain phosphorylation [58,59]. Also an activation of the RhoA/ROCK signaling cascade has been suggested depending upon interleukin-6 and TNFα, two cytokines found to be increased in blood of older human and rodents [19,[59], [60], [61], [62]]. And while others have demonstrated that old TNFα knockout mice are protected from intestinal barrier dysfunction [19] it remains to be determined if the dysregulation of arginase activity and NO homeostasis found in the present study is related to a TNFα-dependent regulation of the RhoA/ROCK/arginase signaling cascade.

## 5 Conclusion

Taken together, our data further bolster the hypothesis that even healthy aging is associated with changes in intestinal microbiota composition and intestinal barrier function. Our data also suggest that the impairments at the level of intestinal barrier may not primarily result from shifts in intestinal microbiota composition. Rather, alterations in intestinal NO homeostasis, and herein specifically an increased activity of arginase and decreased bioavailability of NO in intestinal mucosa, may trigger the loss of intestinal tight junction proteins subsequently leading to an increased translocation of bacterial (endo)-toxins (see Graphical Abstract). However, further studies are needed to determine whether alterations alike are also found in aging humans and if a modulation of the intestinal NO homeostasis has a persisted protective effect on aging associated intestinal barrier dysfunction.

## CRediT authorship contribution statement

Annette Brandt: Formal analysis, Investigation, Writing – original draft, Writing - review & editing. Anja Baumann: Investigation. Angélica Hernández-Arriaga: Formal analysis, Investigation. Finn Jung: Investigation. Anika Nier: Investigation. Raphaela Staltner: Investigation. Dragana Rajcic: Investigation. Christian Schmeer: Methodology, Resources. Otto W. Witte: Methodology, Resources. Barbara Wessner: Methodology, Resources. Bernhard Franzke: Resources, Investigation. Karl-Heinz Wagner: Methodology, Resources. Amélia Camarinha-Silva: Formal analysis, Investigation. Ina Bergheim: Conceptualization, Methodology, Writing – original draft, Writing - review & editing, Supervision, Funding acquisition.

## Funding

The present work was funded by grants from German Research Foundation within the Priority Program SPP1656 (FKZ: BE2376/8-1 to IB, CA1708/1-1 to ACS), the European Union's Horizon 2020 research and innovation programme under the Marie Sklodowska-Curie grant agreement no. 859890 (SmartAge), the Herzfelder Family Foundation/ Austrian Science Fund FWF (P35271 to IB) and the Austrian Science Fund FWF (I04844 to IB).

## Declaration of competing interest

The authors declare that they have no known competing financial interests or personal relationships that could have appeared to influence the work reported in this paper.

## Data Availability

Data are made available upon reasonable request
