# Supplementary material for: Impairments of intestinal arginine and NO metabolisms trigger aging-associated intestinal barrier dysfunction and ‘inflammaging'
Source: Redox Biol. 2022 Oct 31;58:102528. doi: 10.1016/j.redox.2022.102528 (PMC9649383; doi:10.1016/j.redox.2022.102528)
Supplement: Multimedia component 1 [file mmc1.pdf]

Supplemental Table 1. Primer sequences.

	Forward (5´-3´)	Reverse (5´-3´)
18S	GTA ACC CGT TGA ACC CCA TT	CCA TCC AAT CGG TAG TAG CG
Bmi1	TGG GAG TGA CAA GGC CAA CA	ACT GGG GCT GTT GCT GGT TC
Cramp	TGC TCC GAG CTG TGG ATG AC	CAG GGT GAC TGC CCC CAT AC
Cd14	GAG TTG TGA CTG GCC CAG TCA GC	GCA AAA GCC AGA GTT CCT GAC
inos	CCC CTG GAA GTT TCT CTT CAA AGT C	GAT TCT GGA ACA TTC TGT GCT GTC C
Lgr5	GTG GTG GGA AGA CGG AAT CG	AAT GGT CAG GGC CAA CAG GA
Lrig1	ATG GGA GCC AGC TGA AGT CG	CGC GAC AGA CCA TCA AAT GC
Lysozym	GCT GAC TGG GTG TGT TTA GC	TGA TCC CAC AGG CAT TCT TAG
Muc2	TCC TGA CCA AGA GCG AAC AC	ACA GCA CGA CAG TCT TCA GG
Myd88	CCC TAG GGC AGA GGG GAA GA	ATG CCT GTG TGT GCA GAG GAG
p16	CCA AGA GCG GGG ACA TCA AG	AAG AAA AAG GCG GGC TGA GG
Tert	ACC TGC CGA CCT TTC CTT CC	GCC TCT GGC CTC GTT AAG CA
Tlr4	AGC CAT TGC TGC CAA CAT CA	GCT GCC TCA GCA GGG ACT TC
Occ	CAT CAG CCA TGT CCG TGA GG	GGG GCG ACG TCC ATT TGT AG
Zo1	GCA GAC TTC TGG AGG TTT CG	CTT GCC AAC TTT TCT CTG GC

Bmi1: B lymphoma Mo-MLV insertion region 1 homolog; Cd14: cluster of differentiation 14; Cramp: Cathelicidin-related antimicrobial peptide; Defa1: Defensin alpha 1; inos: inducible NO synthase; Lgr5: Leucine-rich repeat-containing G-protein coupled receptor 5; Lrig1: Leucine-rich repeats and immunoglobulin-like domains protein 1; Lyz1: Lysozym 1; Muc2: Mucin-2; Myd88: myeloid differentiation primary response 88; Occ. Occludin; Tert: telomerase reverse transcriptase; Tlr4: Toll-like receptor 4; Zo1: Zonula occludens-1.

Supplemental Table 2: Age and BMI of tested human male individuals.

	Young subjects	Old subjects
**n (male)**	16	16
**age (years)**	27.1 ± 0.7	81.8 ± 1.5*
**height (m)**	1.79 ± 0.01	1.75 ± 0.02
**body weight (kg)**	73.8 ± 2.1	79.1 ± 2.6
**BMI (kg/m^2)**	22.9 ± 0.6	25.9 ± 0.5*

Values are means ± sem. *p<0.05 due to unpaired two-tailed students t-test. BMI: body mass index.

Supplemental Table 3: Effect of aging on intestinal morphology and goblet cells in mice.

	3 months	24 months
**Villus length (µm) - proximal small intestine**	346 ± 12	311 ± 25
**Villus width (µm) - proximal small intestine**	73.0 ± 2.1	80.5 ±1.0*
**Crypt depth (µm) - proximal small intestine**	83.1 ± 2.8	77.4 ± 3.7
**Enterocytes height (µm)**	22.3 ± 0.5	23.7 ± 0.7
**Number of goblet cells/100 µm villus proximal small intestine**	5.0 ± 0.2	5.2 ± 0.3
***Muc2* mRNA – proximal small intestine**	100 ± 15	98.3 ± 15
**Number of goblet cells/100 µm villus distal small intestine**	4.5 ± 0.2	4.4 ± 0.2
**Number of goblet cells/100 µm crypt colon**	7.2 ± 0.5	7.5 ± 0.4

Values are means ± sem. mRNA results are shown as % of younger mice (n=5-9). *$p<0.05$ due to unpaired two-tailed students t-test. Muc2: mucin 2.

Supplemental Table 4. Phyla, families and genera enriched or depleted in proximal small intestine of 3 months and 24 months old mice.

	**3 months**	**24 months**	
**Family**	**average abundance (%)**	**average abundance (%)**	***p*-value**
Bifidobacteriaceae	0.02 ± 0.02	0.35 ± 0.11	0.0059
Erysipelotrichachaceae	2.88 ± 1.01	10.91 ± 2.38	0.0237
**Genus**	**average abundance (%)**	**average abundance (%)**	***p*-value**
Allobaculum	2.77 ± 1.01	10.86 ± 2.38	0.0237
Bifidobacterium	0.02 ± 0.02	0.35 ± 0.11	0.0059
Coprobacillus	0.02 ± 0.00	0.00 ± 0.00	0.0045
Desulfocurvus	0.04 ± 0.01	0.01 ± 0.00	0.0321
Olsenella	0.15 ± 0.04	0.64 ± 0.18	0.0237
Rikenella	0.10 ± 0.03	0.02 ± 0.01	0.0237
Unclassified Lachnospiraceae	2.65 ± 0.41	0.99 ± 0.25	0.0061
Unclassified Porphyromonadaceae	14.74 ± 1.91	6.60 ± 1.04	0.0018

Values are means ± sem. Only significant (p<0.05) differences between the two groups are indicated. Non-parametric Wilcoxon test assuming unequal variances.

Supplemental Table 5: Effect of FMT on markers of body weight, markers of senescence and intestinal permeability, antimicrobial peptides and stem cell markers in old mice.

	o+ yFMT	o+ oFMT
**Body weight (g)**	32.8 ± 1.0	33.2 ± 1.0
**PAI-1 (ng/ml)**	1.6 ± 0.2	1.5 ± 0.1
**ZO-1 protein#**	3.5 ± 0.7	2.7 ± 0.5
***Muc2* mRNA**	100 ± 13	89.1 ± 9.7
***Lysozyme* mRNA**	100 ± 34	93.0 ± 21
***Lgr5* mRNA**	100 ± 21	75.5 ± 10
***Tert* mRNA**	100 ± 15	100 ± 8.4

Values are means ± sem. mRNA results are shown as % of o+yFMT and detected in small intestinal tissue, while PAI-1 levels were detected in plasma (n=6-10). *p<0.05 due to unpaired two-tailed students t-test, #densitometric analysis of protein staining. PAI-1: plasminogen activator inhibitor 1; ZO-1: zonula occludens-1; Muc2: mucin-2; Lgr5: Leucine-rich repeat-containing G-protein coupled receptor 5; Tert: telomerase reverse transcriptase; FMT: fecal microbiome transfer; o: old; y: young.

Supplemental Table 6. Phyla, families and genera enriched or depleted in proximal small intestine of old mice treated with young and old FMT

	**o+oFMT**	**o+yFMT**	
**Closest related species**	**average abundance (%)**	**average abundance (%)**	***p*-value**
Bacteroidales bacterium	10.8 ± 5.4	17.9 ± 6.5	0.037
Muribaculum intestinale	2.0 ± 0.9	3.3 ± 1.5	0.015

Values are means ± sem. Only significant (p<0.05) differences between the two groups are indicated. Non-parametric Wilcoxon test assuming unequal variances. FMT: fecal microbiome transfer, o: old.

Supplemental Table 7. Phyla, families and genera enriched or depleted in old mice treated with and without norNOHA

	o+NaCl	o+norNOHA	
**Genus**	**average abundance (%)**	**average abundance (%)**	***p*-value**
Lactobacillus	9.4 ± 5.0	16.2 ± 5.8	0.04

Values are means ± sem. Only significant (p<0.05) differences between the two groups are indicated. Non-parametric Wilcoxon test assuming unequal variances. norNOHA: N(ω)-hydroxy-nor-L-arginine, o: old.

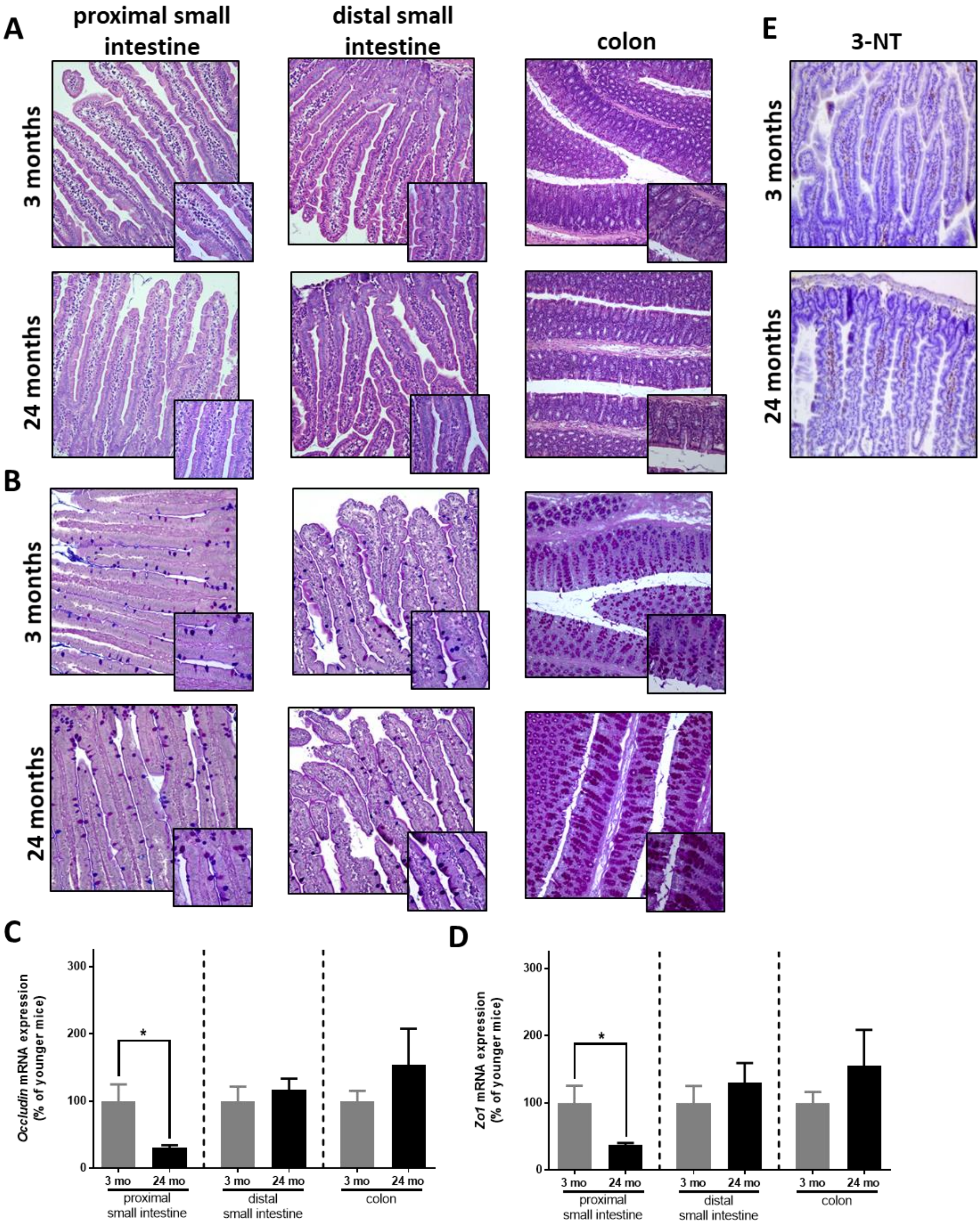

Supplemental Figure 1: Representative pictures (200x, 630x or rather 400x) of (A) H&E staining and (B) goblet cell staining, (C) occludin and (D) Zonula occludens-1 (Zo1) mRNA expression, representative pictures of 3-nitrotyrosine (3-NT) staining in intestinal tissue of 3 months (mo) and 24 months old mice. Values are means ± sem. *p<0.05 due to unpaired two-tailed students t-test.

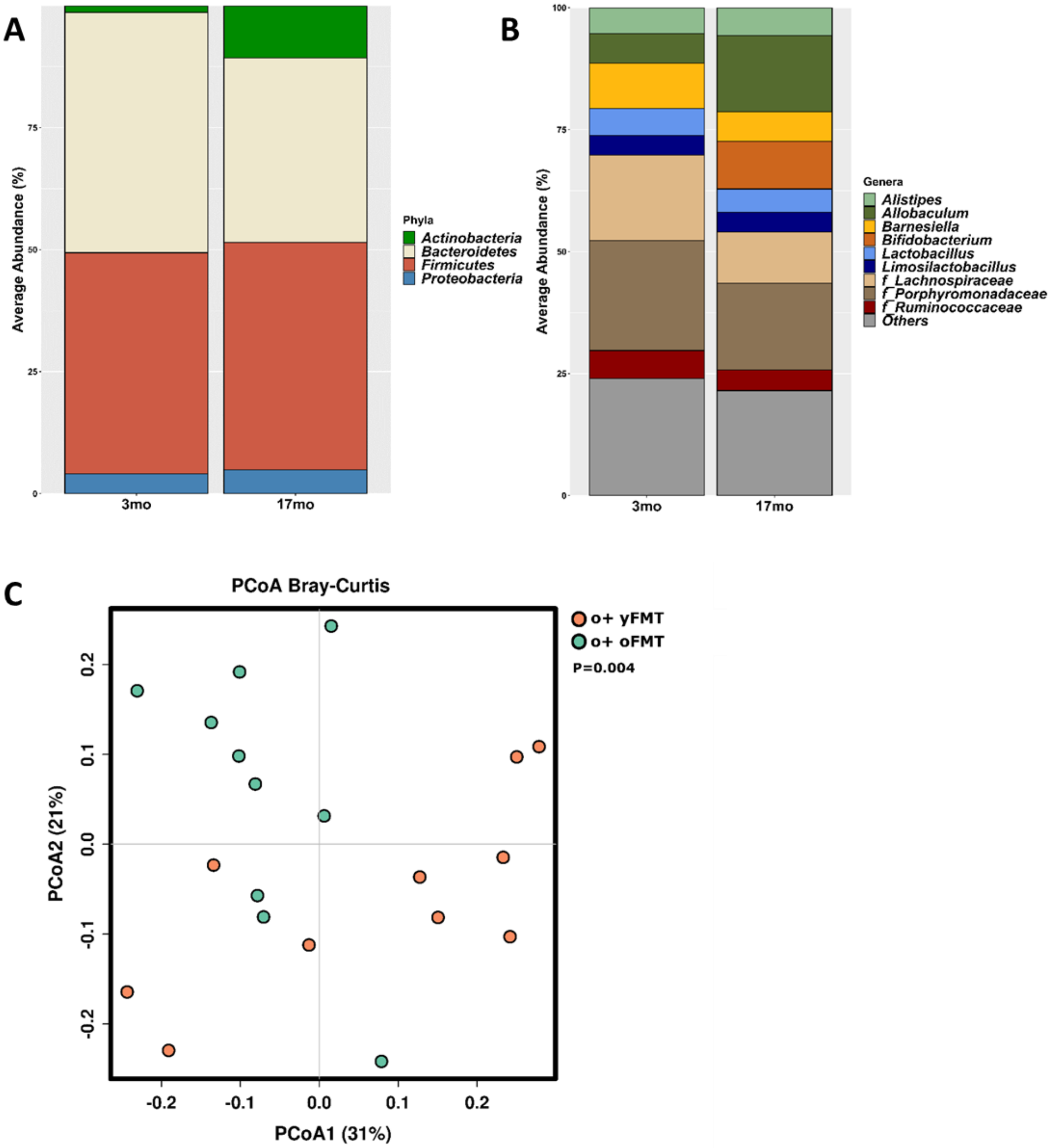

Supplemental Figure 2: (A,B) Analysis of fecal transplant of 3 months and 17 months old mice (A) at phyla level showing the highest average abundance (>0.05%) and (B) at genera level presenting the top 10 genera. (C) Analysis of microbial composition in proximal small intestine of old mice receiving FMT (fecal microbiome transfer) of young mice and of old mice (o+yFMT, o+oFMT). PCoA plot showing the bacterial communities. Each point represents the bacterial ecology of one sample. mo: months.

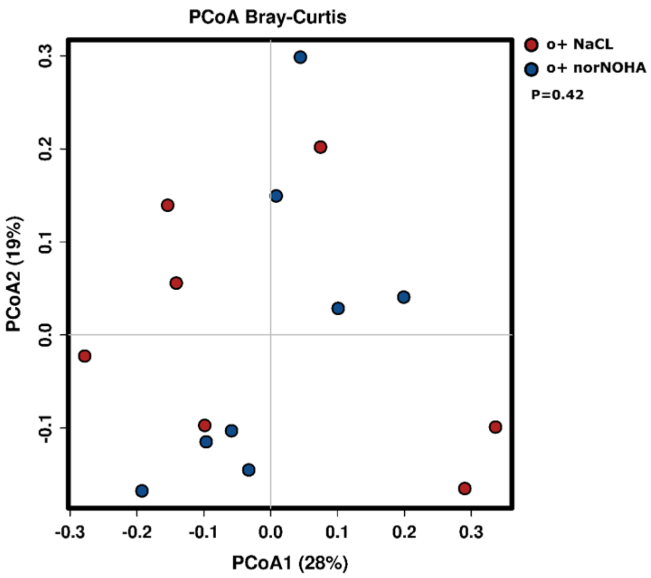

Supplemental Figure 3: Analysis of microbial composition in proximal small intestine of old mice receiving NaCl or norNOHA (N(ω)-hydroxy-nor-L-arginine) i.p.. PCoA plot showing the bacterial communities. Each point represents the bacterial ecology of one sample.
